# Deep brain stimulation does not modulate resting-state functional connectivity in essential tremor

**DOI:** 10.1093/braincomms/fcae012

**Published:** 2024-01-27

**Authors:** Amar Awad, Filip Grill, Patric Blomstedt, Lars Nyberg, Johan Eriksson

**Affiliations:** Umeå Center for Functional Brain Imaging (UFBI), Umeå University, 90187 Umeå, Sweden; Department of Integrative Medical Biology, Physiology Section, Umeå University, 90187 Umeå, Sweden; Umeå Center for Functional Brain Imaging (UFBI), Umeå University, 90187 Umeå, Sweden; Department of Radiation Sciences, Umeå University, 90185 Umeå, Sweden; Department of Clinical Science, Neuroscience, Umeå University, 90185 Umeå, Sweden; Umeå Center for Functional Brain Imaging (UFBI), Umeå University, 90187 Umeå, Sweden; Department of Integrative Medical Biology, Physiology Section, Umeå University, 90187 Umeå, Sweden; Department of Radiation Sciences, Umeå University, 90185 Umeå, Sweden; Umeå Center for Functional Brain Imaging (UFBI), Umeå University, 90187 Umeå, Sweden; Department of Integrative Medical Biology, Physiology Section, Umeå University, 90187 Umeå, Sweden

**Keywords:** essential tremor, deep brain stimulation, caudal zona incerta, resting-state fMRI, functional connectivity

## Abstract

While the effectiveness of deep brain stimulation in alleviating essential tremor is well-established, the underlying mechanisms of the treatment are unclear. Essential tremor, as characterized by tremor during action, is proposed to be driven by a dysfunction in the cerebello-thalamo-cerebral circuit that is evident not only during motor actions but also during rest. Stimulation effects on resting-state functional connectivity were investigated by functional MRI in 16 essential tremor patients with fully implanted deep brain stimulation in the caudal zona incerta during On-and-Off therapeutic stimulation, in a counterbalanced design. Functional connectivity was calculated between different constellations of sensorimotor as well as non-sensorimotor regions (as derived from seed-based and data-driven approaches), and compared between On and Off stimulation. We found that deep brain stimulation did not modulate resting-state functional connectivity. The lack of modulation by deep brain stimulation during resting-state, in combination with previously demonstrated effects on the cerebello-thalamo-cerebral circuit during motor tasks, suggests an action-dependent modulation of the stimulation in essential tremor.

## Teaser

Awad *et al*. used resting-state functional MRI to investigate differences in functional connectivity between On and Off deep brain stimulation in patients with essential tremor, and show that functional connectivity is not modulated by the stimulation. The lack of modulation during resting-state suggests an action-dependent modulation of deep brain stimulation.

## Graphical Abstract

**Graphical Abstract:**
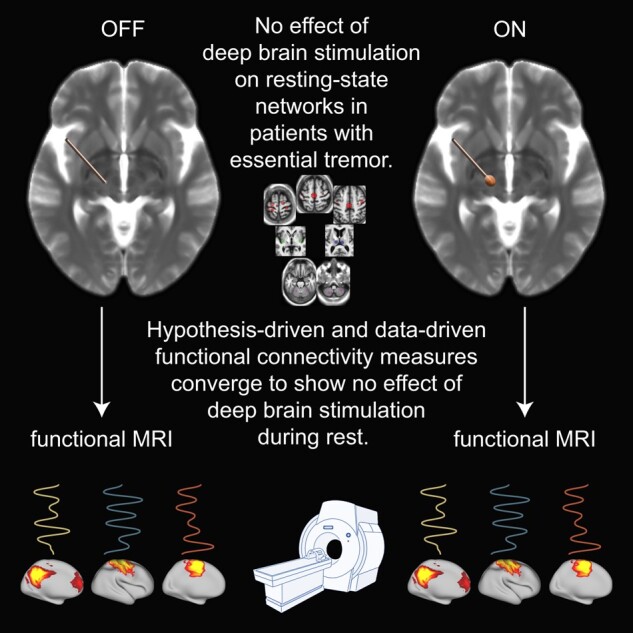

See Stouwe (https://doi.org/10.1093/braincomms/fcae060) for a scientific commentary on this article.

## Introduction

While the effectiveness of deep brain stimulation (DBS) in alleviating essential tremor (ET) is well-established, the underlying mechanisms of the treatment remain unclear. ET, the most common movement disorder, results in bilateral action tremor^1^ and is caused by a dysfunctional cerebello-thalamo-cerebral (CTC) circuit. This dysfunction results in pathological tremor oscillations during movement, but the neuronal activity within the circuit has also been reported to be distorted during rest, without evident tremor.^2,3^ In the current study, we investigate the effects of DBS on the CTC circuit dynamics in ET patients during resting-state.

DBS in the posterior subthalamic area (PSA), including the caudal zona incerta (cZi), and the ventral intermediate thalamic nucleus (Vim) is effective in alleviating tremor.^4-9^ Although DBS is thought to exert its effect by interrupting the propagation of tremor oscillations at the level of the stimulated target (cZi or Vim),^4,5,10^ it has been shown to modulate the neuronal activity of the entire CTC circuit.^11-15^ Indeed, by combining task-based functional MRI (fMRI) with cZi/PSA-DBS during different motor tasks, we showed that DBS resulted in modulation of the sensorimotor CTC circuit blood oxygen level-dependent (BOLD) signal in a complex manner as exhibited by task-depended as well as task-independent effects.^15^ Investigating DBS effects during motor tasks, with and without tremor as in our previous study, was motivated as tremor in ET are present during action and rarely during rest.^1,16^

Notably, functional imaging studies indicate abnormalities in the neuronal activity of the CTC circuit not only during motor tasks but also during rest, when the circuit is not engaged and tremor not present.^3,17^ ET pathophysiology has been examined in several resting-state fMRI (rs-fMRI) studies showing differences in functional connectivity within the CTC circuit as compared to healthy controls.^3,18^ For example, functional connectivity has been shown to be decreased between the cerebellum and the sensorimotor cortex, decreased between primary and premotor sensorimotor cortices and increased between the cerebellum and thalamus.^19-22^ Furthermore, functional connectivity among regions outside the sensorimotor network, such as the default mode and frontoparietal networks, has also been reported to be altered in ET.^23,24^ Whether those alternations in functional connectivity are affected by DBS is still unknown and has not been studied before. Here, we aimed to study cZi-DBS effects on slow BOLD fluctuations as measured by rs-fMRI during On-and-Off therapeutic DBS in ET patients. We predicted that DBS would modulate the functional connectivity within the CTC circuit.

## Material and methods

### Patients and surgical procedure

We included 16 patients with ET (9 male; average age 70 years, range 52–80 years) and fully implanted DBS in the cZi/PSA. Out of 60 ET patients with cZi DBS at our department, 35 were excluded mainly due to MR-incompatible DBS systems, but other reasons included cognitive impairment/dementia, claustrophobia, or significant head and resting tremor. Of the remaining 25 patients, 17 consented but one died from unrelated causes before the initiation of the study. ET diagnosis was set by a movement-disorders specialist according to the “consensus statement of the Movement Disorder Society on Tremor”.^25^ A new consensus on the classification of tremor^1^ was established after the diagnosis of our patients and the conduction of the study, but this was not deemed to change the diagnosis of the included patients. See Table 1 for patient demographics, DBS parameters, tremor severity, and improvement. In summary, all patients had severe action tremor and seven patients had mild tremor at rest.

**Table 1:** Patient demographics and stimulation parameters

Patient	Sex	Age	Active DBS during fMRI	Family history	Disease duration	Months since surgery	Stimulation parameters^a^	Tremor during OFF DBS^b^	Tremor during ON DBS^b^	Improvement
1	m	75	Right	Yes	57 yrs	27	2.3 V, 60 µs, 130 Hz	20	1	95%
2	f	78	Left	No	11 yrs	70	2.7 V, 60 µs, 150Hz	21	3	86%
3	f	78	Left	Yes	16 yrs	36	1.2 V, 60 µs, 160 Hz	19	1	95%
4	f	80	Left	Yes	30 yrs	54	1.3 V, 60 µs, 140 Hz	27	2	93%
5	f	59	Right	Yes	46 yrs	43	1.5 V, 60 µs, 130 Hz	15	1	93%
6	m	67	Left	Yes	50 yrs	17	1.8 V, 60 µs, 160 Hz	15	1	93%
7	m	78	Left	Yes	23 yrs	34	1.6 V, 60 µs, 140 Hz	8	0	100%
8	f	75	Left	Yes	15 yrs	37	2.3 V, 60 µs, 140 Hz	25	2	92%
9	m	67	Left	No	50 yrs	36	1.8 V, 60 µs, 160 Hz	16	2	88%
10	f	69	Left	Possibly	11 yrs	11	1.5 V, 60 µs, 140 Hz	18	1	94%
11	m	68	Left	Yes	33 yrs	44	1.6 V, 60 µs, 140 Hz	15	0	100%
12	f	70	Left	Yes	ca 50 yrs	59	1.8 V, 60 µs, 150 Hz	16	2	88%
13	m	75	Left	No	20 yrs	59	2.2 V, 60 µs, 160 Hz	19	2	89%
14	m	57	Left	Yes	ca 40 yrs	26	2.3 V, 60 µs, 160 Hz	21	0	100%
15	m	52	Left	Yes	12 yrs	50	2.5 V, 60 µs, 140 Hz	24	7	71%
16	m	77	Left	No	42 yrs	17	1.7 V, 60 µs, 140 Hz	23	1	96%

^a^Amplitude, pulse width, and frequency.

^b^ETRS contralateral hand tremor and function, item 5 or 6 and 11–14 (25 points in total).

**Figure 1:**
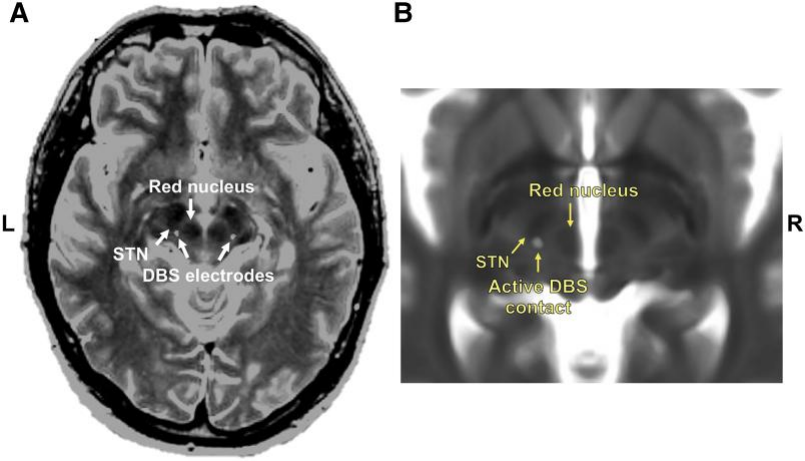
**The surgical DBS target and active contact.** (**A**) A pre-operative axial MR-image fused with a postoperative CT for a representative patient, demonstrating the localization of the tips of the DBS electrodes in the caudal zona incerta (cZi) within the the posterior subthalamic area (PSA); posteromedial to the subthalamic nucleus (STN) at the level of the maximal diameter of the red nucleus. (**B**) The mean location of the active DBS contacts is 1.4 mm below the anterior commissure-posterior commissure line (AC-PC) level. The electrode artefact is averaged from group-specific T1-weighted images and superimposed on a T2-weighted image in Montreal Neurological Institute (MNI) space.

The implantation of the electrodes was done under general anaesthesia without microelectrode recording or intraoperative test stimulation. The target in the cZi/PSA was visually identified on a stereotactic T2-weighted MRI slightly posteromedial to the posterior tip of the subthalamic nucleus (STN) at the level of the maximal diameter of the red nucleus (Fig. 1A). The location of the electrodes was verified using an intraoperative, or postoperative, CT fused with the pre-operative MRI. The patients were implanted with electrode model 3389 Medtronic and a single ‘implanted pulse generator’ (Activa, Medtronic).

The patients had been receiving chronic DBS in the cZi/PSA with a stable clinical response for at least 1 year (range 1–5.8 years). The mean location of the active cathodic contact was 12.5 ± 1.4 mm lateral, 6.6 ± 1.1 mm behind, and 1.4 ± 1.4 mm below the midcommissural point (MCP) (Fig. 1B). The average improvement in contralateral hand tremor and function (item 5 or 6 and 11–14) according to the essential tremor rating scale (ETRS) was 91% (18.9 ± 4.7 during Off stimulation as compared to 1.6 ± 1.7 during on stimulation). All patients gave written informed consent, and the study was approved by the local medical ethical board and was performed in accordance with the Declaration of Helsinki.

### DBS–MRI interaction, fMRI data acquisition, and experimental design

Only patients with MR-compatible/conditional DBS systems could be recruited for this study. Moreover, due to safety concerns, mostly related to the risk of heating at the tip of the electrode, we adhered to a strict MR imaging protocol that included lower magnetic field strength (1.5T), use of transmit/receive radiofrequency head coil, and adjusted imaging parameters to keep the head-specific absorption rate values <0.1 W/kg. The protocol we used was based on knowledge about DBS–MRI interactions at the time of data collection.^26-28^

All scans were performed with a Philips Achieva dStream 1.5T MR scanner. During each DBS condition (On and Off), three experiments/runs were collected: task-based fMRI with different motor tasks as previously published in Awad *et al*.,^15^ rs-fMRI (this study), and task fMRI with a working memory task.^29^ Functional echo-planar imaging (EPI) rs-fMRI runs were performed with the following parameters: 31 interleaved axial slices at a TR 3000 ms, TE 50 ms, flip angle 90°, voxel size 3.44 × 3.49 × 4.4 mm, 0.5 mm inter-slice gap, field of view (FOV) 220 × 220 mm, and matrix size 64 × 63. Axial T1-weighted structural scan was collected after the first functional session with the following acquisition parameters: 180 slices, no inter-slice gap, 1 × 1 × 1 mm voxel size, TR 7.4 s, TE 3.4 ms, flip angle 8°, FOV 256 × 232 mm, and matrix size 256 × 232.

Two rs-fMRI time-series were collected per patient, one for each stimulation condition (unilateral On and Off cZi-DBS). The first five volumes were discarded prior to each session to allow fMRI signal equilibrium. For each acquisition, 154 volumes (∼ 8 minutes) per session were collected. The patients were instructed to lie still in the scanner with their eyes opened and focusing on a fixation cross presented on a screen that was seen via a double-mirror mounted on the head coil.

Therapeutic unilateral left-sided DBS was used in all, except two, patients (who had right-sided DBS activated during the on session). For patients implanted with bilateral DBS electrodes (*n* = 5), the right electrode (ipsilateral to the tested arm during the motor task-experiment) was switched off during the whole experiment. The initial stimulation setting was counterbalanced across patients, i.e. half of the patients started the first session with DBS Off and the other half with DBS On. Therapeutic stimulation parameters were used during the study. These parameters were previously optimized for maximal tremor reduction without side effects.

There was a washout period of ∼25–30 minutes between the On and Off sessions which was deemed sufficient to exclude potential rebound effects (temporary increase in tremor severity above the pre-operative baseline immediately after switching the stimulation Off).^30^ This period included the time when the systems were switched from Off to On (or On to Off), and running two other fMRI experiments (motor and working memory tasks^15,29^). Overt rebound (about 5 minutes) was known to exist in one patient (patient 15) who, consequently, started the experiment with Off stimulation and DBS was turned Off >30 minutes before the first fMRI session.

The head movements were restricted by using foam padding between the head and head coil in all patients. To further restrict head movements, bite bars fixed on the head coil were used when tolerated (six patients). These bite bars were custom-made for each patient to match the patient’s own teeth before the scanning session.

### Statistical analysis

#### Image processing

Image data (T1 and EPI volumes) from the two patients with active right-sided electrodes during the session were flipped with respect to the mid-sagittal plane before pre-processing.

##### Pre-processing of fMRI data

fMRI data were pre-processed using CONN toolbox version 20b based on SPM12 implemented in MATLAB. Images were realigned, unwarped, and slice-time corrected. Outlier volumes were detected using the artefact detection tools (ART) as implemented in CONN and by using the option for conservative threshold; an image was defined as an outlier if the head displacement in the *x*-, *y-*, or *z*-direction was greater than 0.5 mm from the previous frame, or if the global mean intensity of an image was greater than 3 SD from the mean image intensity for the entire resting scan. The images were then normalized to the standard Montreal Neurological Institute (MNI) space and smoothed with an 8-mm full-width at half-maximum Gaussian kernel. Functional and structural T1-weithted images were segmented into grey matter, white matter and CSF. The hardware-related artefacts resulted in signal loss most pronounced at the electrode tip and the extension cables sited over the left parietal cortex. Affected voxels are excluded from group analysis by implicit masking.

##### Denoizing

fMRI data were further denoized by using component-based noise correction method (*CompCor*) implemented in CONN. Twelve realignment parameters and their quadratic effects (Friston24-parameters), potential outlier scans, and signals from white matter and cerebrospinal fluid masks were used as confounds. Further, the data were bandpass-filtered (0.008–0.09 Hz). Global signal regression was not applied.

A group-specific anatomical template was created from the individual 16 T1-weighted images using DARTEL (diffeomorphic anatomical registration through exponentiated lie algebra) for a more precise intersubject alignment.^31^ A group-specific anatomical image was created by averaging individual normalized T1-weighted, which then was used to visualize the group-specific electrode artefacts and as an anatomical background on which fMRI results were projected.

#### fMRI data analysis

##### Identifying the sensorimotor network and creating sensorimotor regions of interest (ROIs)

**Figure 2:**
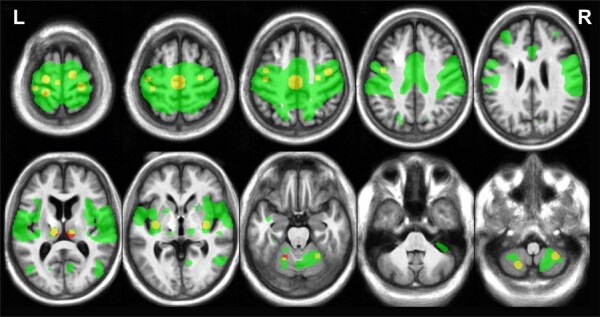
**Sensorimotor network and ROIs.** The sensorimotor functional connectivity map as extracted from voxelwise correlation to Yeo-17 left-motor-cortex ROI (green map), and the created sensorimotor ROIs (red/yellow circles).

The sensorimotor circuit was defined based on seed-to-voxel functional connectivity with the Yeo-17 left-motor-cortex ROI.^32^ We chose this ROI because it generated a more inclusive/complete sensorimotor network (especially regarding the left hemisphere where DBS was turned On and Off during the experiment) as compared to other tested seeds, see Supplementary Fig. 1. Across both rs-fMRI runs (during On and Off DBS) of each patient, the Pearson’s correlation between fMRI time-series at each voxel and the left-motor-cortex ROI was computed. The resultant functional connectivity map is shown in Fig. 2 at *P* < 0.0001 uncorrected (exploratory *post hoc* analysis). Sensorimotor ROIs were created as spheres around relevant peak coordinates with a 5 mm radius, except for the supplementary motor area where 10 mm was used to include both hemispheres. The ROIs included the primary motor cortex, premotor cortex, supplementary motor area, postcentral gyrus, thalami, putamen, cerebellum lobule V/VI and cerebellum lobule VIII), see Fig. 2.

The analysis of cZi-DBS effects on slow BOLD fluctuations as measured by rs-fMRI during On-and-Off therapeutic DBS in ET patients was done in four steps, as further detailed below.

##### Calculation of functional connectivity between averaged sensorimotor ROIs

**Figure 3:**
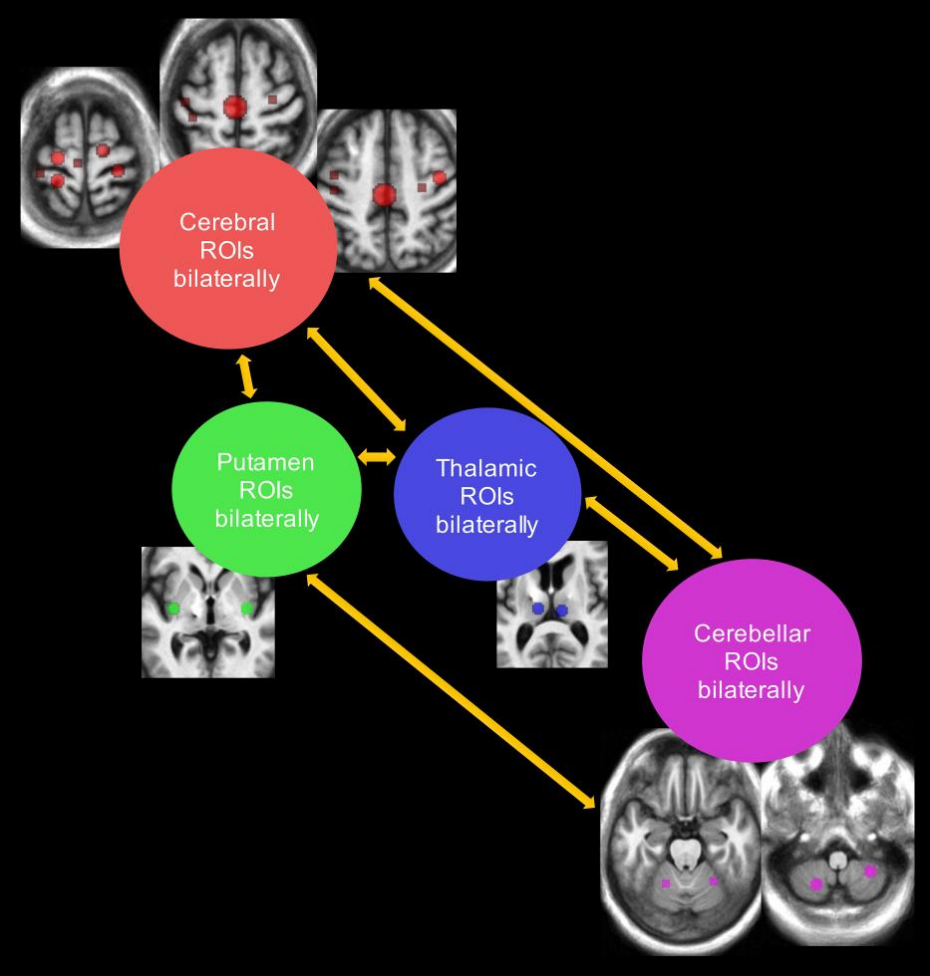
**Calculation of functional connectivity between averaged sensorimotor ROIs.** The four grouped sensorimotor ROIs and their six connections were used to calculate wide-spread bilateral functional connectivity differences between On and Off DBS.

To examine widespread and bilateral functional connectivity differences between the cerebral cortex, cerebellum, putamen and cerebellum, ROIs from these locations were grouped and treated as one ROI. That resulted in four grouped ROIs: cerebral cortex regions bilaterally, bilateral thalamic regions, bilateral putamen regions, and all cerebellar regions bilaterally; see Fig. 3. Cross-correlation was computed between extracted average time-series from grouped ROIs during On and Off sessions. Correlation coefficients were then Fisher transformed to *z-*values. Paired-sample *t*-tests were used to calculate correlation value differences between On and Off DBS.

##### Calculation of functional connectivity between sensorimotor ROIs separately for each hemisphere

Cross-correlations were computed between time-series from each ROI in one cerebral hemisphere with time-series from cerebellar ROIs on the opposite side, which resulted in 21 tested connections for each hemisphere. Correlation coefficients were then Fisher transformed to *z*-values. Paired-sample *t*-tests were used to calculate correlation value differences between On and Off DBS. This analysis is more sensitive than the previous step since BOLD signal is not averaged across regions. It is motivated by the lateralized anatomical connections through crossing fibres between the cerebellum and cerebrum. Further, only unilateral DBS was active in this study which prompted examination of each hemisphere separately.

##### Calculation of amplitude of low frequency fluctuations within sensorimotor ROIs (grouped and separated)

The power spectrum of each ROI was obtained by transforming the ROI time-series to the frequency domain. The mean square root of the power in the frequency range across 0.01–0.1 Hz was used as a measure of amplitude of low frequency fluctuations (ALFF).^33^ The ALFF values were calculated for grouped and separated ROIs, similar to the two previous steps in functional connectivity analysis. The procedure was performed on DBS Off and On fMRI runs separately. The ALFF score for each ROI and fMRI run was then entered into a paired *t*-test to investigate differences in ALFF as a function of DBS. Several rs-fMRI studies have shown altered ALFF values in the CTC circuit in ET patients.^34-36^

##### Dual-regression to investigate DBS effects on resting-state networks identified through independent component analysis

Dual-regression independent component analysis (ICA) was conducted in 14 patients (excluding the two with active right-sided DBS during fMRI), and followed the steps suggested by Nickerson *et al*.^37^ Prior to denoizing, fMRI runs from both Off and On sessions as well as for each individual were concatenated and entered into a group ICA restricted to 30 components. The resulting group-average components were visually inspected and identified according to known resting-state networks: visual, frontoparietal, lateral sensorimotor, default mode, salience, medial sensorimotor, cerebellar and ventral attention network. The remaining 22 networks represented noise such as movement artefacts, BOLD signal from white matter and ventricles. Each group-average network was then regressed into each subject’s and condition’s time resolved dataset giving subject and condition specific time-series. The network specific time-series where then regressed into the same time-resolved dataset yielding subject and condition specific functional connectivity maps. The resulting connectivity maps were then entered into a paired-samples *t*-test using FSL’s randomize function^38^ to look for differences between DBS On and Off (5000 permutations, threshold-free cluster enhancement (TFCE) corrected). This step is motivated by rs-fMRI studies indicating functional connectivity abnormalities in resting-state networks beyond the sensorimotor circuit.^23,24^

## Results

The average framewise displacements as calculated according to the approach suggested by Power *et al*.^39^ did not differ between Off and On conditions (Off: 0.14 ± 0.09 mm, On: 0.16 ± 0.10 mm, *P* = 0.52, paired-sample *t*-test).

### DBS differences in functional connectivity between averaged sensorimotor ROIs

**Figure 4:**
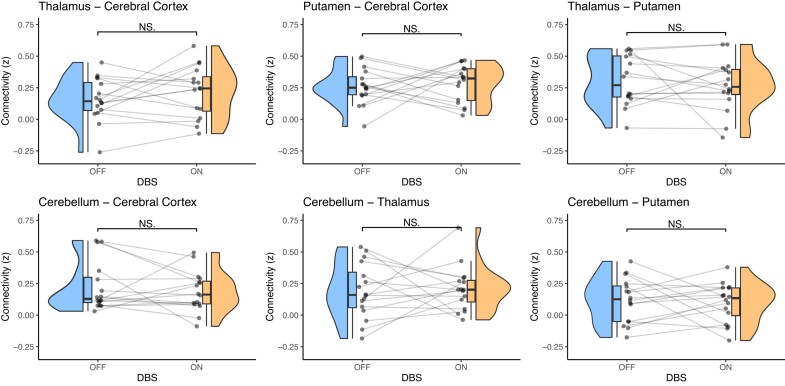
**Functional connectivity between averaged sensorimotor regions.** Raincloud plots illustrate the relatively similar distributions in connectivity values between On and Off DBS. Connectivity/correlational values (*z*) are shown on the *y*-axis. The distribution of connectivity and individual connectivity values for each patient and DBS setting are depicted alongside boxplots representing median connectivity and interquartile range. NS. = not significant (paired-sample *t*-tests).

There was no statistically significant difference in correlation values between On and Off DBS when calculating the differences in functional connectivity between averaged/grouped bilateral sensorimotor ROIs (all *P*’s > 0.24 paired-sample *t*-test, uncorrected), see Fig. 4.

### DBS differences in specific functional connectivity between sensorimotor ROIs separately for each hemisphere

No statistically significant difference was detected in correlation values between On and Off DBS when calculating differences in functional connectivity between separate sensorimotor ROIs within each hemisphere (all *P* > 0.09 paired-sample *t*-test, uncorrected), see Supplementary Fig. 2.

### DBS differences in ALFF within sensorimotor ROIs (averaged and separated)

The ALFF values in sensorimotor ROIs, both averaged and separated, did not differ significantly between On and Off DBS (all except one test with *P* > 0.13 paired-sample *t*-test, uncorrected). There was a difference in the ALFF value in the left dorsal premotor cortex ROI with *P* = 0.03, which until replicated in an independent sample, should be considered non-significant given many tests without correction for multiple comparisons.

### DBS differences in multiple resting-state network as calculated via dual-regression ICA

**Figure 5:**
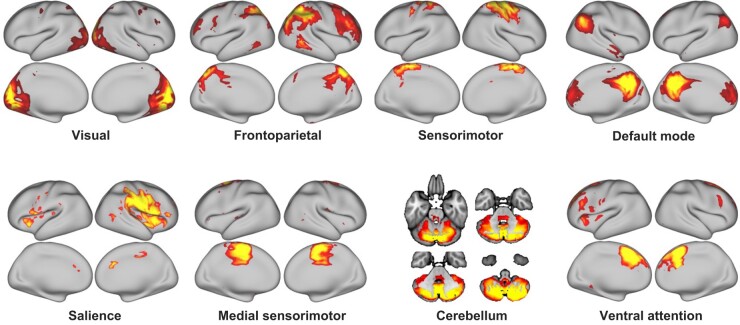
**Resting-state networks as identified by ICA.** Eight networks/components could be mapped to canonical resting-state networks. The remaining 22 components reflected noise (not shown here).

Using ICA, eight networks were identified as shown in Fig. 5. Dual-regression analysis did not show a statistically significant difference between DBS On and Off (TFCE-corrected, *P* > 0.05) in any of the eight identified components.

## Discussion

We investigated cZi-DBS effects on slow BOLD fluctuations as measured by rs-fMRI during On-and-Off therapeutic DBS in ET patients and found no significant modulation of resting-state functional connectivity. This was the case when examining DBS effects on (i) widespread functional connectivity between sensorimotor cerebral cortex, thalamus, putamen, and cerebellum; (ii) hemisphere-specific functional connectivity in ROIs within the aforementioned regions; (iii) ALFF within sensorimotor ROIs; and (iv) multiple well-known resting-state networks, sensorimotor as well as non-sensorimotor. Thus, the correlation in BOLD signal fluctuations among nodes, within and outside the CTC circuit, is comparable in Off and On DBS.

Since this is the first study to examine the effects of DBS on functional connectivity in ET, a comparison with other studies is difficult. However, the null-findings of this study could be compared to findings from previous reports about the effects of vim-thalamotomy on functional connectivity. Recent studies showed differences in functional connectivity after focused ultrasound thalamotomy. For example, functional connectivity was shown to increase between the thalamus and premotor cortex,^40^ and within the sensorimotor and visuospatial networks^41^ after, as compared to before, thalamotomy. Differences in functional connectivity following thalamotomy might be related to distinct mechanisms of action for lesioning as compared to electrical stimulation. Moreover, in contrast to our study which measures acute changes in functional connectivity due to DBS, thalamotomy studies assessed changes months (3–6 months) after the procedure. Thus, functional connectivity alternations may reflect long-term changes in resting-state networks due to thalamotomy.

Regarding stereotactic radiosurgical thalamotomy with Gamma knife, Tuleasca *et al.* investigated rs-fMRI functional connectivity in ET patients before^42,43^ and after^44,45^ thalamotomy in a number of publications with overlapping populations and findings, They reported several rs-fMRI networks before or after unilateral thalamotomy to be correlated with tremor reduction postoperatively.^42-44^ However, they applied ICA for the identification of resting-state networks and included all or most of the resultant networks, including some that might represent noise from motion or respiration artefacts, white matter and ventricles.^42,43^ Also, the altered connectivity patterns reported by Tuleasca and colleagues were mainly based on correlations with tremor reductions, while we have here refrained from using correlations due to the small sample size.^46^ In summary, potential differences in mechanisms of action for lesioning and methodological limitations of the aforementioned studies explain why thalamotomy, but not DBS, might affect the functional connectivity in ET.

Although being one of the largest DBS-fMRI studies, the sample size of this study is still small, and the study might simply be underpowered to detect potential effects of interest. However, the distributions of ROI-ROI correlation values are relatively similar during On and Off DBS (Fig. 4 and Supplementary Fig. 2), which implies that modest effects would still be hard to find even with a much larger sample size. Moreover, negative findings were demonstrated despite (deliberately) liberal statistical testing, and are thus unlikely to represent false negative findings.

The choice of ROIs was based on their functional specifications.^47^ They represented well-known nodes within the sensorimotor network (Fig. 2). Therefore, we consider it unlikely that the choice of ROIs impacted the results negatively. Also, functional connectivity and ALFF were probed with different constellations of connections, from averaged-ROI-connections to capture potential widespread changes, to individual ROI-connections to capture potential specific changes between ROIs. Moreover, the dual-regression analysis was based on ICA, which is a data-driven method for identifying networks independent of the choice of ROIs.^37,48^

MR-signal drop-out due to the DBS hardware is another potential limitation. The metallic objects in the DBS electrodes and extension cables are known to result in susceptibility artefacts (signal loss) mostly pronounced around the electrode tip and the left parietal cortex.^14,49,50^ Obviously, this precluded the acquisition of useful image data from those areas which affected the lateral part of the sensorimotor network, and the salience network that seemed to be right-dominated. Importantly, most of the sensorimotor circuit (Fig. 2) and other circuits (Fig. 5) are not disturbed by signal drop-out. Due to safety concerns which necessitated strict inclusion criteria and MR imaging protocol (addressed in detail in the ‘DBS–MRI interaction section’), the data quality and signal-to-noise ratio were compromised. Despite these limitations, our data were of sufficient quality to generate a well-known sensorimotor network based on ROI-to-voxel functional connectivity, and moreover, ICA could identify the canonical resting-state networks (Figs. 2 and 5).

The present finding that DBS did not affect resting-state functional connectivity within and outside the sensorimotor circuit can be related to our previous observation of DBS effects on functional brain activity.^15^ In that previous study, differences in BOLD-signal amplitude during DBS On versus Off were assessed for a postural holding task, a pointing task, and a resting control task. The main result was that DBS-On led to reduced activity in the primary sensorimotor cortex and cerebellum (lobule VIII) during the postural task but not during rest. This observation is in good agreement with the present finding of no DBS effects during resting state. In addition, in Awad *et al*.^15^ it was found that DBS-On led to increased activity in left premotor cortex during all tasks (the postural and pointing tasks as well as rest), and even to selective increases in activity during the rest condition in the supplementary motor area and the cerebellum (lobule IV/V). One obvious explanation of why DBS effects were seen on functional brain activity at rest but not on resting-state functional connectivity concerns the different analytic approaches. Task effects capture transient modulation of blood flow and BOLD signal, whereas functional connectivity might reflect stable functional networks of regions that typically are co-activated and minimally influenced by brief interventions. By this view, modulation of the BOLD-signal amplitude during rest by DBS could reflect elements of motor preparedness/planning and task-set switching (i.e. getting prepared for the upcoming postural holding task and task set switching from the pointing task to rest)^51,52^ that are not taxed during a long period of rest in rs-fMRI. Thus, the modulation of BOLD signal during rest as well as motor tasks in Awad *et al*.^15^ might reflect multiple aspects of action, and we therefore propose that DBS modulation of the sensorimotor circuit in ET is action-dependent in a broad sense. This notion is coherent with the fact that DBS alleviates tremor, which in ET is action tremor that is present during action and rarely during rest.^1,16^

## Conclusions

In this study, DBS effects on functional connectivity in ET were probed by rs-fMRI during On-and-Off therapeutic DBS in the cZi/PSA. DBS did not modulate resting-state functional connectivity in sensorimotor or non-sensorimotor networks. As DBS previously was shown to modulate the cerebello-cerebral circuit during motor tasks,^15^ we argue that DBS modulation is action-dependent in ET.

## Supplementary Material

fcae012_Supplementary_Data

## Data Availability

All data supporting the conclusions of this manuscript will be made available by the authors upon request to any qualified researcher.
